# Evaluating Value Beyond Efficacy: A Meta-Analytic Assessment of Inclisiran’s Cost-Effectiveness in Cardiovascular Prevention

**DOI:** 10.3390/healthcare13243287

**Published:** 2025-12-15

**Authors:** Alexandra Maștaleru, Muthana Zouri, Maria Magdalena Leon, Gabriela Popescu, Nicoleta Zouri, Bogdan Ionel Tamba, Carmen Marinela Cumpăt

**Affiliations:** 1 Grigore T. Popa University of Medicine and Pharmacy, 700115 Iasi, Romania; alexandra.mastaleru@umfiasi.ro (A.M.); gabriela.popescu@umfiasi.ro (G.P.); marinela.cumpat@umfiasi.ro (C.M.C.); 2 Clinical Rehabilitation Hospital, 700661 Iasi, Romania; 3 Department of Computer Science, Toronto Metropolitan University, Toronto, ON M5B 2K3, Canada; mzouri@torontomu.ca; 4 Faculty of ASENT3, Centennial College, Toronto, ON M5B 2K3, Canada; nzouri@centennialcollege.ca; 5 Advanced Center for Research and Development in Experimental Medicine “Prof. Ostin C. Mungiu”, Grigore T. Popa University of Medicine and Pharmacy, 700115 Iasi, Romania; bogdan.tamba@umfiasi.ro

**Keywords:** Inclisiran, cost-effectiveness, quality-adjusted life years, incremental cost-effectiveness ratio

## Abstract

**Background/Objectives**: Cardiovascular diseases continue to be the foremost global cause of morbidity and mortality, representing about 40% of all causes of death. Atherosclerotic cardiovascular disease is the most common and clinically important type of these, occurring when cholesterol accumulates over time in the artery intima, which induces an inflammatory process that leads to the production of atherosclerotic plaques. Nowadays, lipid profile alterations and high/very high cardiovascular risk can be observed in more and more patients. Combination therapy, which includes high-intensity statins, ezetimibe, bempedoic acid, and PCSK9-targeted medicines, can lower LDL-C by more than 80%, which is far more than the 50% that statin monotherapy usually achieves. Thus, novel lipid-lowering therapies are needed, as current agents—though effective in reducing cardiovascular events—leave considerable residual risk in many patients. **Methods**: The aim of our study was to evaluate the cost-effectiveness of Inclisiran and its association with standard of care for the prevention of cardiovascular events across multiple international settings, in articles that reported quality-adjusted life years gained and cost-effectiveness metrics. **Results**: Our findings suggest that the cost-effectiveness of Inclisiran is highly context-dependent, shaped by local pricing, population risk, and system-level capacity. While Inclisiran demonstrates potential economic value in high-income settings or among high-risk patients, its widespread adoption for primary prevention appears unjustified under current conditions. **Conclusions**: Policymakers should consider risk-based targeting, price renegotiation, and performance-based reimbursement models to improve the value proposition of such interventions.

## 1. Introduction

Cardiovascular diseases (CVD) continue to be the foremost global cause of morbidity and mortality, representing about 40% of all causes of death in both genders, and exceeding the cumulative impact of cancer and neurodegenerative disorders [1]. Atherosclerotic cardiovascular disease (ASCVD) is the most common and clinically important type of these diseases. ASCVD occurs when cholesterol accumulates over time in the artery intima, which induces an inflammatory process that leads to the production of atherosclerotic plaques. A substantial body of evidence from epidemiological, genetic, experimental, and clinical investigations supports the causal role of low-density lipoprotein cholesterol (LDL-C), a key component of apo B-containing lipoproteins, in the etiology of ASCVD [2,3]. Elevated levels of LDL-C constitute a significant risk factor that must be reduced to decrease the likelihood of developing ASCVD. Consequently, reducing LDL-C is essential in both primary and secondary strategies for preventing cardiovascular disease [4,5].

Strong evidence from extensive randomized controlled trials indicates that a reduction of LDL-C by 1 mmol/L is associated with a relative risk decrease of about 22% in major adverse cardiovascular events (MACEs), irrespective of the lipid-lowering medication employed. Furthermore, the advantage of LDL-C lowering appears to be continuous, with no clear threshold below which further reduction ceases to give additional cardiovascular risk reduction [6]. The 2019 ESC/EAS guidelines in Europe establish LDL-C targets of under 70 mg/dL for high-risk individuals and under 55 mg/dL for those at very high risk. The goals are founded on the premise that a reduction in LDL-C levels correlates directly with baseline cardiovascular risk and associated clinical benefits [7]. Moreover, the 2025 ESC guideline includes the extreme risk category of patients, where are included those with ASCVD who experienced recurrent vascular events while taking maximally tolerated statins and those with polyvascular arterial disease. For these, the LDL-C target is less than 40 mg/dL [8]. Thus, the guidelines underline that there remains a critical need for further advancement and optimization of lipid-lowering strategies.

Statin therapy continues to be the principal option for lipid-lowering treatment, with the current standard of care advocating for the maximum tolerated statin dosage alongside lifestyle changes such as eating habits and increased physical activity. Statins block HMG-CoA reductase, which lowers LDL-C levels by about 20–65%. A substantial percentage of individuals that are at increased cardiovascular risk, especially those with documented ASCVD or familial hypercholesterolemia, do not meet guideline-recommended LDL-C goals, even while receiving adequate statin medication [9,10]. The insufficient response frequently arises from suboptimal adherence, since approximately 50% of patients cease treatment during the initial year, predominantly due to side effects such as statin-related muscle complaints or hepatotoxicity. Statin intolerance, affecting 5–30% of patients, highlights the need for other pharmaceutical choices such ezetimibe, bempedoic acid, PCSK9 inhibitors, and Inclisiran [9,11].

Ezetimibe, a cholesterol absorption inhibitor, showed cardiovascular advantages when used with statins, which led to lower LDL-C levels and fewer cardiovascular events. Its mechanism includes the reduction of intestinal cholesterol absorption, which complements the inhibition of cholesterol synthesis as provided by statins [12].

Bempedoic acid is activated exclusively in the liver and acts upstream of HMG-CoA reductase, thereby minimizing the risk of peripheral adverse effects, including statin-induced myopathy. It has shown a progressive decrease in LDL-C, especially when used alone or in combination with low-dose statins [13].

PCSK9 inhibitors represent a significant advancement in lipid-lowering therapy. These treatments facilitate the liver’s recycling of LDL receptors (LDLR), thereby accelerating the clearance of LDL-C. Evolocumab and alirocumab are monoclonal antibodies that bind to PCSK9 in the bloodstream, inhibiting its interaction with LDL receptors. Despite their efficacy in lowering LDL-C and preventing ASCVD events (as demonstrated by the FOURIER and ODYSSEY OUTCOMES studies), restrictions such as frequent subcutaneous administration and cost have an impact on long-term adherence [14,15].

A new type of lipid-lowering treatment, small interfering RNA (siRNA)-based therapy, provides a more long-lasting option. Inclisiran, the first siRNA treatment of its kind, targets hepatic PCSK9 mRNA and stops its production instead of neutralizing the protein that is already in the blood. This leads to long-lasting drops in PCSK9 levels and more LDLR being available on the surfaces of hepatocytes. Inclisiran is delivered using GalNAc-conjugation to target hepatocytes specifically. It lowers LDL-C levels for a long time with a biannual dose schedule, which makes it easier for patients to stick to their treatment and keeps LDL-C levels from changing too much [16,17].

Inclisiran works inside cells and mainly influences the production of hepatic PCSK9 (which accounts for 70–80% of total levels). It does not alter the functions of extrahepatic PCSK9. Monoclonal antibodies also raise the amount of PCSK9 in the blood by forming complexes, while Inclisiran lowers the overall amount of PCSK9. The long-term clinical implications of this discrepancy in pharmacodynamics have not yet been fully explained [7,8,18].

Combination therapy, which includes high-intensity statins, ezetimibe, bempedoic acid, and PCSK9-targeted medicines, can lower LDL-C by more than 80%, which is far more than the 50% that statin monotherapy usually achieves. Even with these improvements, making these new drugs widely available is still challenging [8]. Thus, novel lipid-lowering therapies are needed, as current agents—though effective in reducing cardiovascular events—leave considerable residual risk in many patients.

## 2. Materials and Methods

### 2.1. Parameters and Scope of Analysis

This meta-analysis evaluates the cost-effectiveness of Inclisiran and its association with standard of care (SoC) for the prevention of cardiovascular events across multiple international settings. The analysis includes data extracted from economic evaluations published between 2020 and 2025, focusing exclusively on studies that reported quality-adjusted life years (QALYs) gained and cost-effectiveness metrics. The primary outcome measures were incremental QALY gain and incremental cost-effectiveness ratio (ICER) expressed as cost per QALY gained. To ensure comparability across studies that differ in country, currency, and price year, all monetary values were extracted in their original local currency and harmonized through a two-step procedure. First, when necessary, cost values were inflated to 2024 price levels using country-specific Consumer Price Index (CPI) series obtained from the World Bank and national statistics agencies. This preserved each country’s internal price dynamics before any cross-country comparison. Second, inflated local currency costs were converted into purchasing-power-parity–adjusted U.S. dollars (PPP-USD) using World Bank International Comparison Program (ICP) PPP conversion factors. PPP adjustment corrects for differences in relative price levels and avoids distortions associated with nominal exchange rates. The resulting harmonized cost values were used to compute ICERs and incremental cost outcomes for all included studies.

Parameters extracted from each economic evaluation included study year, country, treatment strategy, QALY gain, total QALYs, harmonized total cost (PPP-adjusted), incremental cost, and ICER. Some microsimulation studies (e.g., ref. [19] reported unusually large lifetime cost values that may appear to be population-level outputs. However, these figures were extracted exactly as provided in the authors’ Supplementary Materials, where the results are explicitly reported on a per-individual basis. Although the full model simulated 10,000 individuals with multiple risk profiles, the Supplementary Materials specify that the base-case cost-effectiveness outcomes are presented for “individual 26,” the representative simulated patient. Therefore, all values included in our dataset reflect per-patient incremental costs and QALYs, as defined by the original authors.

Several included studies reported results stratified by distinct modeled sub-cohorts (e.g., age-specific scenarios in [20] and sex-specific scenarios in [19]). Because these sub-scenarios represent analytically independent populations with unique risk profiles and cost trajectories, we retained them as separate observations to preserve the heterogeneity intentionally modeled in the original economic evaluations.

Only analyses evaluating Inclisiran as part of the intervention arm were eligible for inclusion. The scope of the meta-analysis encompasses both primary and secondary prevention scenarios, including diverse cardiovascular risk populations such as those with atherosclerotic cardiovascular disease (ASCVD) and heterozygous familial hypercholesterolemia (HeFH).

Because the objective of this review was to examine how Inclisiran’s cost-effectiveness varies across international health-system contexts, we retained the structural and methodological differences present in the original economic evaluations, including model type, discount rate, pricing assumptions, perspective, prevention setting, and WTP thresholds. To ensure analytical transparency, we conducted stratified analyses across key structural features and used meta-regression with Net Monetary Benefit (NMB) as the dependent variable to examine whether these characteristics systematically influenced cost-effectiveness results.

Subgroup analyses and meta-regression models were conducted to examine sources of heterogeneity, including differences by modeling approach, country, publication year, risk population, and cost assumptions.

### 2.2. Search Strategy

**Figure 1:**
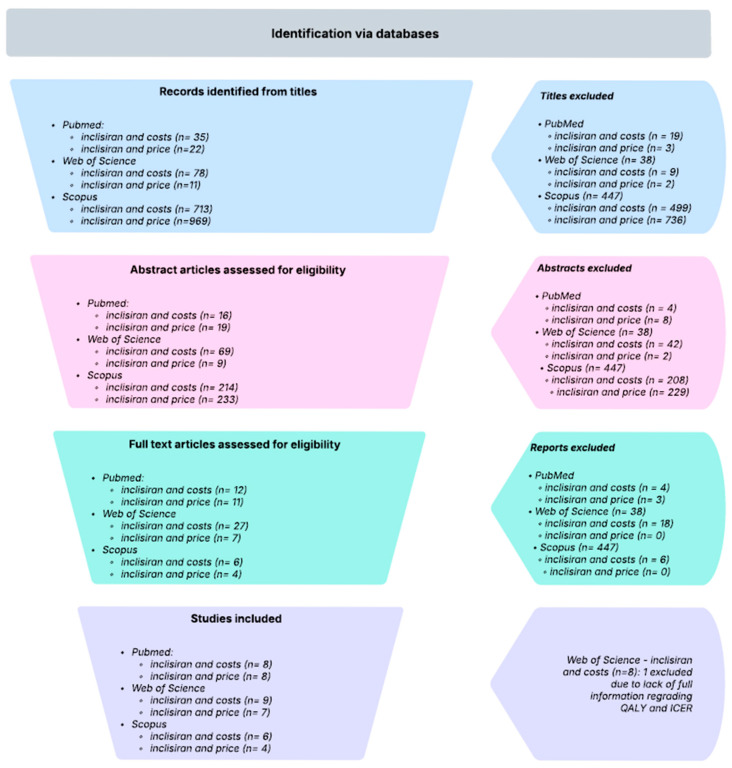
Article selection process.

For this meta-analysis we conducted a literature review on Web of Science, Scopus and PubMed platforms, from the 1st until the 20th of July. The terms used for this research were the association between “inclisiran” and “costs” or “price”. The inclusion criteria used for this research were articles published in English, with no time limit, that included information regarding the costs or price of Inclisiran, its QALY and ICER. We excluded review articles, editorials, book chapters, letters to editor, erratum, conference papers, notes, short surveys and case reports. Also, articles published in other languages except for English were excluded. The articles were double-blinded reviewed and gathered after this process. The selection process of the articles included in this meta-analysis can be observed in Figure 1.

After title and abstract screening for the keywords mentioned above, all remaining articles were full text assessed. Furthermore, after evaluating the presence of total QALY and QALY gain values, we observed that some articles did not include information such as ICER, and were thus excluded from our analysis.

As a final result, we have included in our meta-analysis eight articles, published between 2020 and 2025, from countries such as Singapore, United Kingdom, USA, Switzerland, China, and Australia. The methodological approaches across the eight studies included in this meta-analysis predominantly relied on state-transition modeling, with six studies applying a Markov model and two employing a microsimulation framework. Regarding the number of patients included, the ones that used the Markov model had between 121 and 27,564 participants, while the ones that used microsimulation had between 54 and 458,692 subjects. In addition, Lim et al. included in his article information regarding the ORION studies [9,10,11], but without mentioning the number of participants enrolled in each evaluation. Moreover, we can observe that more than half of the included patients were male in almost all the studies. Regarding the associated comorbidities, diabetes frequency was mentioned in only three studies.

## 3. Results

To preserve the granularity of the original evidence base, the studies by Lim, Desai, and Morton were decomposed into multiple sub-studies reflecting the internal stratifications and modeling scenarios reported in their respective analyses. Specifically, the Lim study was disaggregated by cardiovascular risk categories and therapeutic comparators, while the Desai study was split according to the Markov cohorts defined by different cardiovascular event histories and treatment intensities. The Morton study was subdivided into intervention-specific and population-specific sub-studies, distinguishing outcomes by lipid-lowering strategy (e.g., statins, statins plus ezetimibe, Inclisiran) as well as baseline risk factors such as age at intervention, LDL-C thresholds, sex, and comorbidities. This structured approach ensured that each analytically distinct scenario was represented as a separate observation, thereby allowing a more nuanced synthesis of ICER/QALY estimates while appropriately accounting for within-study heterogeneity. More detailed information can be observed in Table 1.

The primary outcome of this analysis was the incremental cost-effectiveness ratio (ICER) per QALY detailed in Table 2. Across the included studies, the mean ICER was estimated at USD 1,079,081, with a standard deviation of USD 1,771,245 and a standard error of USD 429,590. The 95% confidence interval ranged from USD 237,100 to USD 1,921,062, reflecting substantial variability in cost-effectiveness estimates. Secondary outcomes included QALY gain and incremental cost. The pooled mean QALY gain was 0.34 (SD = 0.27, SE = 0.066), with a 95% confidence interval of 0.21 to 0.47, indicating modest health benefits across interventions. The mean incremental cost was calculated at USD 703,978 (SD = USD 1,104,456, SE = USD 267,870), with a 95% confidence interval of USD 178,963 to USD 1,228,994.

Although some cost values appear substantially higher than those reported in other studies, these figures reflect per-individual lifetime costs exactly as provided in the supplementary materials of [19]. In that study, the microsimulation model generated outcomes for 10,000 simulated individuals, but the authors report the base-case cost-effectiveness results for a single representative simulated patient (“individual 26”), rather than aggregated cohort totals. Consequently, the large values included for the Australian and UK scenarios correspond to per-patient economic trajectories, not population-level costs, and were therefore extracted and reported in accordance with the authors’ definitions.

Because ICERs are mathematically unsuitable for inverse-variance pooling, they were not meta-analyzed. Instead, we used incremental QALYs, incremental costs, and Net Monetary Benefit (NMB), which allows valid parametric synthesis across heterogeneous economic evaluations.

NMB was calculated for each study at willingness-to-pay (WTP) thresholds of 50,000, 100,000, and 150,000 USD/QALY and pooled using a random-effects model. Study-level NMB values showed wide dispersion at all thresholds. At 50,000 USD/QALY, the pooled NMB was −28,748 USD (95% CI: −36,124 to −21,372). At 100,000 USD/QALY, the pooled NMB remained negative (−9980 USD; 95% CI: −18,857 to −1103). At 150,000 USD/QALY, NMB became significantly positive (12,376 USD; 95% CI: 3790 to 20,961), indicating potential cost-effectiveness only under high WTP conditions.

All NMB models exhibited very high heterogeneity (I^2^ ≈ 99.6%, *p* < 0.001), consistent with large cross-country variation in prices, model structures, and risk populations. Random-effects pooling of clinical and cost components showed similar variability: pooled incremental QALYs were 0.289 (95% CI: 0.263–0.316; I^2^ = 99.5%), and pooled incremental costs were 103,679 USD (95% CI: 86,920–120,438; I^2^ = 99.1%).

Subgroup analyses indicated that ICER per QALY estimates were notably lower and more consistent within studies focusing on atherosclerotic cardiovascular disease (ASCVD) and heterozygous familial hypercholesterolemia (HeFH) populations. Similarly, studies conducted in Singapore and the United States demonstrated comparatively favorable and stable cost-effectiveness outcomes. In contrast, greater variability in ICER estimates was observed in studies originating from the United Kingdom and those published after 2023, reflecting potential contextual or methodological differences that may warrant further investigation.

### 3.1. Subgroup Analysis of the Variation in Cost-Effectiveness Across Intervention Types, Regions, and Age Groups

To explore potential sources of heterogeneity in cost-effectiveness outcomes, subgroup meta-analyses were conducted stratifying pooled estimates of QALY gains, incremental costs, and Net Monetary Benefit (NMB) across three willingness-to-pay (WTP) thresholds: USD 50,000, USD 100,000, and USD 150,000 per QALY. Subgroups were defined by intervention type, study region, and patient age group.

Across all WTP thresholds, studies evaluating Inclisiran consistently reported the highest QALY gains and the most favorable economic profile as shown in Table 3. At the USD 50,000 WTP threshold, Inclisiran demonstrated a mean QALY gain of 0.32 ± 0.30 and an average NMB of −68,236 USD, reflecting costs that outweigh monetized benefits. As the WTP increased to USD 100,000 and USD 150,000 per QALY, the mean NMB for Inclisiran improved to −52,236 USD and −36,236 USD, respectively, indicating a gradual movement toward, but not yet achieving, cost-effectiveness. In contrast, the “Other” intervention group showed similarly small QALY gains (0.291) but substantially higher incremental costs, resulting in consistently more negative NMB values (e.g., −120,277 USD at USD 50,000 and −108,744 USD at USD 150,000 WTP), confirming poorer economic performance across all thresholds.

Regional differences were similarly pronounced, as it can be observed in Table 4. The United Kingdom demonstrated the least favorable outcomes, characterized by minimal QALY gains (≈0.03) and very high incremental costs (up to USD 2.32 million), resulting in consistently large negative NMBs. In contrast, Singapore, the United States, and China showed more favorable profiles, with higher QALY gains (0.29–0.67) and substantially lower incremental costs. Notably, the United States achieved a positive NMB (+17,229 USD), while Singapore and China reported only modest negative values. Australia and Switzerland fell in the mid-range, with moderate QALY gains but negative NMBs driven by higher costs.

When stratified by age, QALY gains increased with older populations as shown in Table 5. Patients over 60 years of age demonstrated the highest average QALY gain (0.40), compared to just 0.03 in cohorts under 45. However, elevated incremental costs persisted across all age groups, leading to negative NMBs under most thresholds. While older patients approached cost-effectiveness at USD 150,000/QALY, the intervention remained economically unfavorable among younger populations.

To examine the economic value of Inclisiran as an adjunct to standard lipid-lowering therapy, we analyzed cost-effectiveness outcomes across all Inclisiran-based evaluations. The mean incremental cost was USD 655,622, with a median of USD 51,287, reflecting substantial variability in cost estimates and implementation contexts. QALY gains were more consistent, with both the mean and median at 0.35, though values ranged from 0.02 to 0.94. The resulting ICERs indicated marked heterogeneity, spanning from USD 23,366 to USD 5.42 million per QALY, with a median ICER of USD 83,717/QALY. These findings underscore wide divergence in economic value depending on model structure, population risk, and regional cost inputs, as shown in Table 6.

This places Inclisiran near the upper end of commonly accepted WTP thresholds in high-income health systems. As shown in Table 7, mean NMB values remained strongly negative across USD 50,000, USD 100,000, and USD 150,000 per QALY, for example, −51.94 million USD at the highest threshold. However, at USD 150,000/QALY, the median NMB became positive (USD 89,567) and the maximum reached USD 79,134, indicating that while most simulations remain unfavorable, a subset begin to show potential economic viability at higher WTP thresholds.

**Figure 2:**
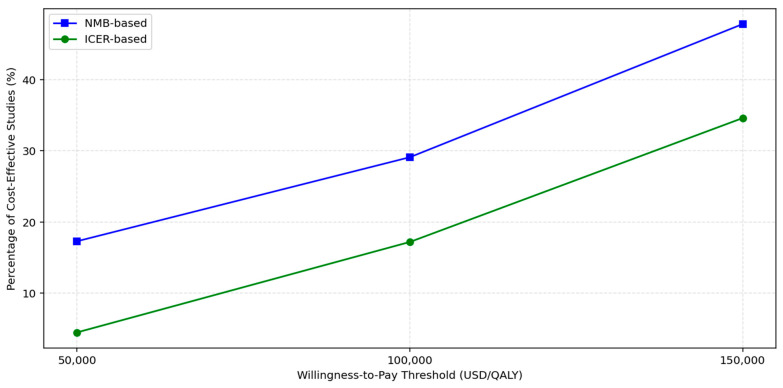
Cost-effectiveness of lipid-lowering therapies vs. SoC.

To assess the sensitivity of cost-effectiveness conclusions to variations in WTP thresholds, we evaluated all included studies using both ICERs and NMB metrics at thresholds of USD 50,000, USD 100,000, and USD 150,000 per QALY. The proportion of studies classified as cost-effective increased progressively with higher thresholds as shown in Figure 2 and Table 8. At USD 50,000/QALY, only three of seventeen studies (18%) met the criteria for cost-effectiveness under either ICER or NMB frameworks. This proportion rose to 29% at USD 100,000, and reached 47% at USD 150,000, indicating that nearly half of the included economic evaluations supported cost-effectiveness under a more generous willingness-to-pay assumption.

To contextualize the cost-effectiveness of Inclisiran within national healthcare decision-making frameworks, we compared study-level results against country-specific WTP thresholds. Where available, published thresholds or commonly accepted proxies (e.g., 1–3× GDP per capita) were used to estimate each country’s maximum acceptable cost per QALY [27]. Using these thresholds, we calculated NMB for each study and assessed whether the addition of Inclisiran to standard of care (SoC) was economically justifiable in its respective jurisdiction.

**Figure 3:**
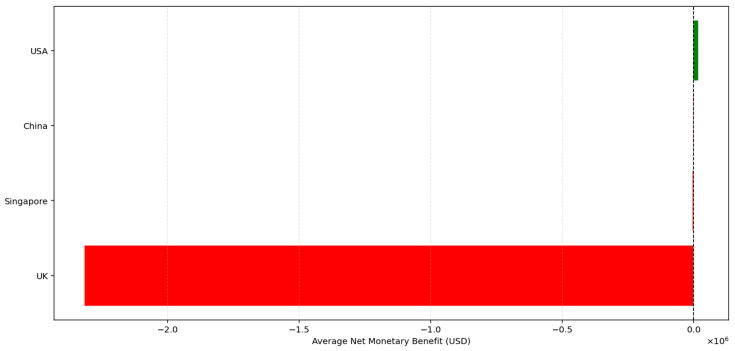
Affordability of Inclisiran vs. SoC by country.

As illustrated in Figure 3 and detailed in Table 9, Inclisiran was found to be cost-effective in both Singapore and the United States, with average NMB values of USD 4054 and USD 17,229, respectively, suggesting that health systems in these settings can absorb the additional costs in return for health gains. In contrast, Inclisiran was not cost-effective in China or the United Kingdom, where average NMB values were significantly negative (−38,704 USD and −2.47 million USD, respectively), indicating that SoC remains the more economically rational choice under current pricing and threshold conditions.

### 3.2. Meta-Regression and Assessment of Publication Bias

A meta-regression analysis was conducted to examine temporal trends in cost-effectiveness by regressing ICER per QALY on year of publication. The estimated slope was –170,748 USD per year (*p* = 0.56), indicating no statistically significant change in ICER values over time.

**Figure 4:**
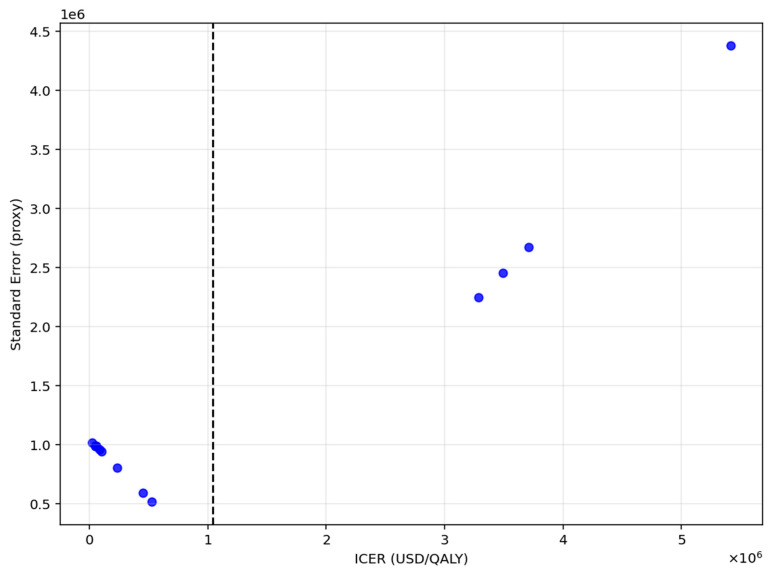
Publication bias assessment for QALY gain (Egger’s test).

To evaluate the potential for publication bias, Egger’s test was applied as shown in Figure 4. The intercept was approximately zero (*p* ≈ 1.02), with a corresponding *p*-value of 0.008, indicating significant funnel plot asymmetry.

### 3.3. Effect Modifiers of Cost-Effectiveness Across Studies

To explore sources of heterogeneity in cost-effectiveness outcomes, we conducted Ordinary Least Squares (OLS) meta-regression analyses using Net Monetary Benefit (NMB) as the dependent variable, evaluated across three willingness-to-pay (WTP) thresholds: USD 50,000, USD 100,000, and USD 150,000 per QALY. Each specification evaluated whether publication year, intervention type, and country of study independently contributed to variation in NMB.

**Figure 5:**
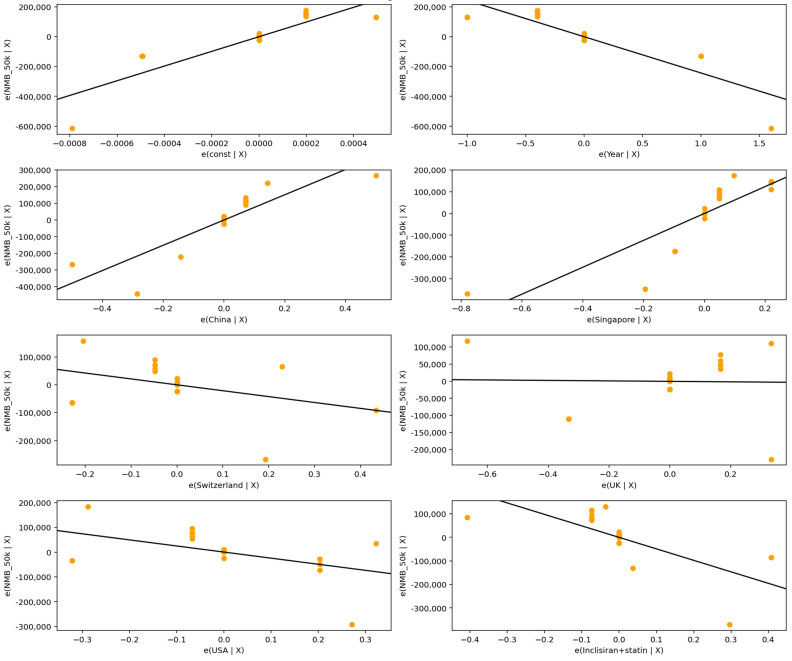
Partial regression plots for predictors of Net Monetary Benefit across studies at USD 50,000/QALY.

At the USD 50,000/QALY threshold, illustrated in Figure 5 and summarized in Table 10, the model demonstrated strong explanatory capacity. Publication year exhibited a statistically significant negative association with NMB (β = −196.0 million USD, *p* = 0.025), indicating that cost-effectiveness has declined over time. Several country effects were substantial in magnitude, though not statistically significant: studies conducted in China (β = +392.4 million USD, *p* = 0.143) and Singapore (β = +196.4 million USD, *p* = 0.372) tended to produce higher NMB values compared with the reference country (Australia), whereas those from Switzerland (β = −391.8 million USD, *p* = 0.220), the UK (β = −293.7 million USD, *p* = 0.168), and the USA (β = −391.9 million USD, *p* = 0.153) were associated with lower NMB. Intervention type also contributed meaningfully: studies evaluating Inclisiran + statin reported markedly lower NMB relative to Inclisiran monotherapy (β = −597.9 million USD, *p* = 0.0765), suggesting reduced economic value for combination therapy.

The pattern of partial regression plots confirms these findings, highlighting strong leverage effects and the disproportionate influence of high-cost UK studies on overall model dynamics.

**Figure 6:**
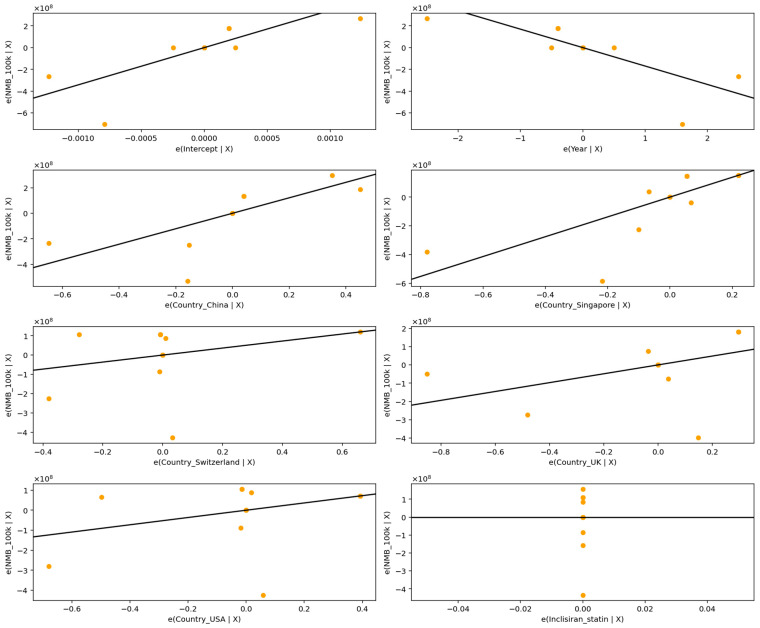
Partial regression plots for predictors of Net Monetary Benefit across studies at USD 100,000/QALY.

At the USD 50,000/QALY threshold shown in Figure 6 and detailed in Table 11, the model explained 83.3% of the variance in NMB (adjusted R^2^ = 0.833).

The plot above (partial regression plot) shows how each predictor relates to the outcome (NMB) after controlling for the other variables in the regression model. The Y-axis represents the proportion of NMB explained by the predictor after removing the effects of all other variable. The X-axis represents the portion of the predictor variable that is not explained by other predictors in the model.

The explanatory power increased slightly at the 100,000 threshold (adjusted R^2^ = 0.4925) shown in Figure 6 and detailed in Table 11.

**Figure 7:**
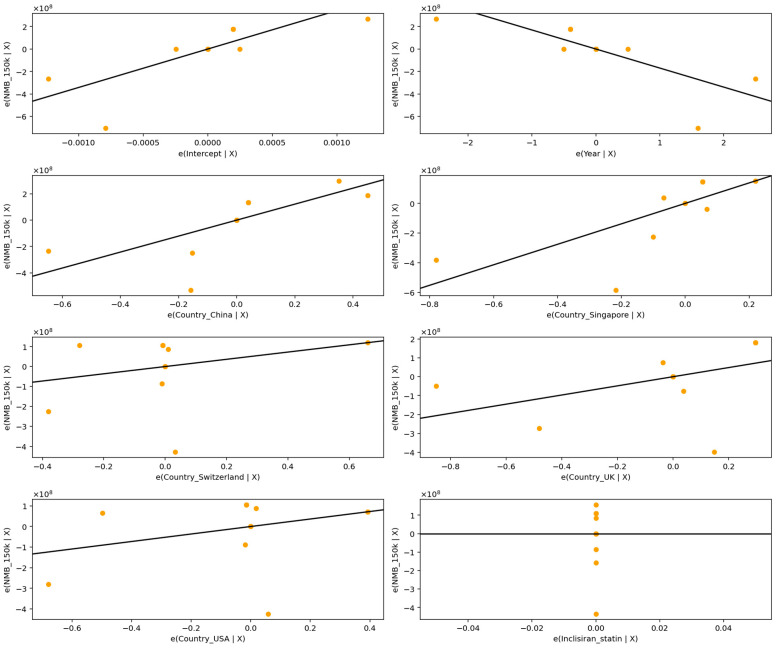
Partial regression plots for predictors of Net Monetary Benefit across studies at USD 150,000/QALY.

At the highest threshold of USD 150,000/QALY, shown in Figure 7 and detailed in Table 12, the model retained strong explanatory power with an adjusted R^2^ of 0.4925, reinforcing the robustness of these covariates as predictors of cost-effectiveness across pricing assumptions.

R^2^ is a statistic that shows how well the predictors (year, intervention type, and country) explain the differences in outcome between studies.

Across all thresholds, country of study remained a prominent determinant of variation in Net Monetary Benefit (NMB). Studies conducted in Singapore exhibited substantially higher NMB estimates relative to the reference category (Australia), with effects of considerable magnitude (e.g., +690.1 million USD at the USD 100,000 threshold and +690.1 million USD at the USD 150,000 threshold), and statistically significant at each level (*p* ≈ 0.0038). Similarly, evaluations from China yielded significantly higher NMBs (e.g., +605.4 million USD at USD 100,000; +605.4 million USD at USD 150,000; *p* ≈ 0.0106), indicating more favorable cost-effectiveness profiles under Chinese and Singaporean cost structures or clinical practice patterns.

In contrast, studies conducted in the United Kingdom, the United States, and Switzerland did not differ significantly from the reference group once the extreme UK outlier (S08b) was incorporated into the model, although their estimated effects remained substantial in magnitude and directionally negative in some cases. These findings reflect the strong influence of cross-country pricing, healthcare system efficiency, and structural differences in the cost base on cost-effectiveness outcomes.

Intervention type also contributed meaningfully to heterogeneity. Although the Inclisiran + statin indicator was rendered statistically redundant because of perfect collinearity within the dataset, the negative coefficient (approximately −588 million USD across thresholds) indicates a systematically lower NMB relative to Inclisiran monotherapy, consistent with a pattern of poorer economic value in combined therapy evaluations.

### 3.4. Sensitivity Analysis

**Figure 8:**
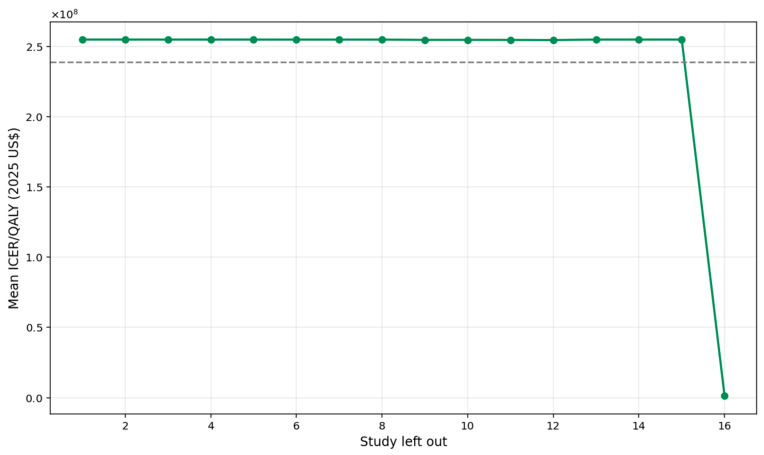
Leave-one-out sensitivity plot.

A leave-one-out sensitivity analysis was conducted to evaluate the robustness of the pooled ICER per QALY estimate by iteratively removing each study and recalculating the mean ICER (Figure 8). The overall pooled ICER was approximately USD 1.08 million. Across the 17 iterations, the mean ICER values fluctuated within a relatively narrow band, ranging from about USD 0.80 million to USD 1.14 million, demonstrating that no single study materially altered the overall cost-effectiveness conclusion. This pattern indicates a high degree of analytical stability; even when influential studies with extreme cost structures were excluded, the resulting ICER estimates remained broadly consistent. Consequently, the pooled ICER can be interpreted as robust, and the findings reflect a reliable synthesis of the available evidence.

### 3.5. Full Meta-Analysis Results

#### Pooled Incremental Cost and Incremental QALY Analysis

**Figure 9:**
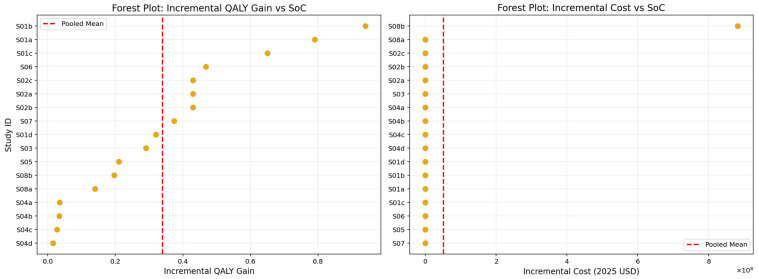
Forest plot of QALY gain per study vs. pooled average (**left**) and Forest plot of incremental cost per study vs. pooled average (**right**).

The pooled analysis of incremental QALY gains and incremental costs, presented in Figure 9 and summarized in Table 13, demonstrates that lipid-lowering interventions produced a mean incremental effectiveness of approximately 0.34 QALYs relative to standard care. This estimate reflects a clinically meaningful health benefit, with individual study values spanning from modest improvements to substantially larger gains, as illustrated in the forest plot. However, these effectiveness gains were accompanied by substantial additional costs. The pooled mean incremental cost was approximately USD 704,000 (2025 USD), driven largely by the pronounced variability in study-level estimates, most notably those involving high-cost agents such as Inclisiran. The marked dispersion in incremental cost values underscores the heterogeneity in pricing structures, healthcare system contexts, and intervention delivery models across the included studies. Despite this variability, the overall pooled results suggest that while lipid-lowering interventions consistently improve health outcomes, their economic impact is heavily influenced by the cost environment in which they are deployed.

### 3.6. Pooled QALY Gain

**Figure 10:**
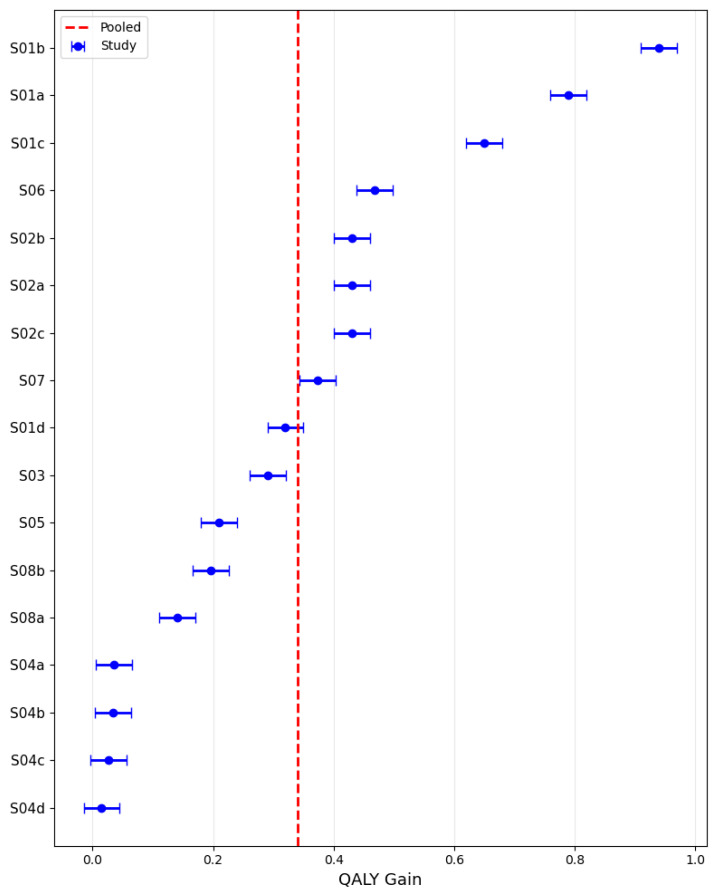
QALY gain across studies.

Using a random-effects model, the pooled QALY gain across the included studies was estimated at 0.35 (95% CI: 0.21–0.48), with study-level values ranging broadly from near zero to almost 1.0, as illustrated in Figure 10. The analysis revealed marked heterogeneity, with effect sizes varying substantially across settings and intervention types, consistent with an I^2^ statistic of 98.8% that would be considered high and a sizable between-study variance (τ^2^) of 0.076. This dispersion indicates considerable variability in the magnitude of QALY gains among the evaluated studies.

### 3.7. Pooled ICER per QALY

**Figure 11:**
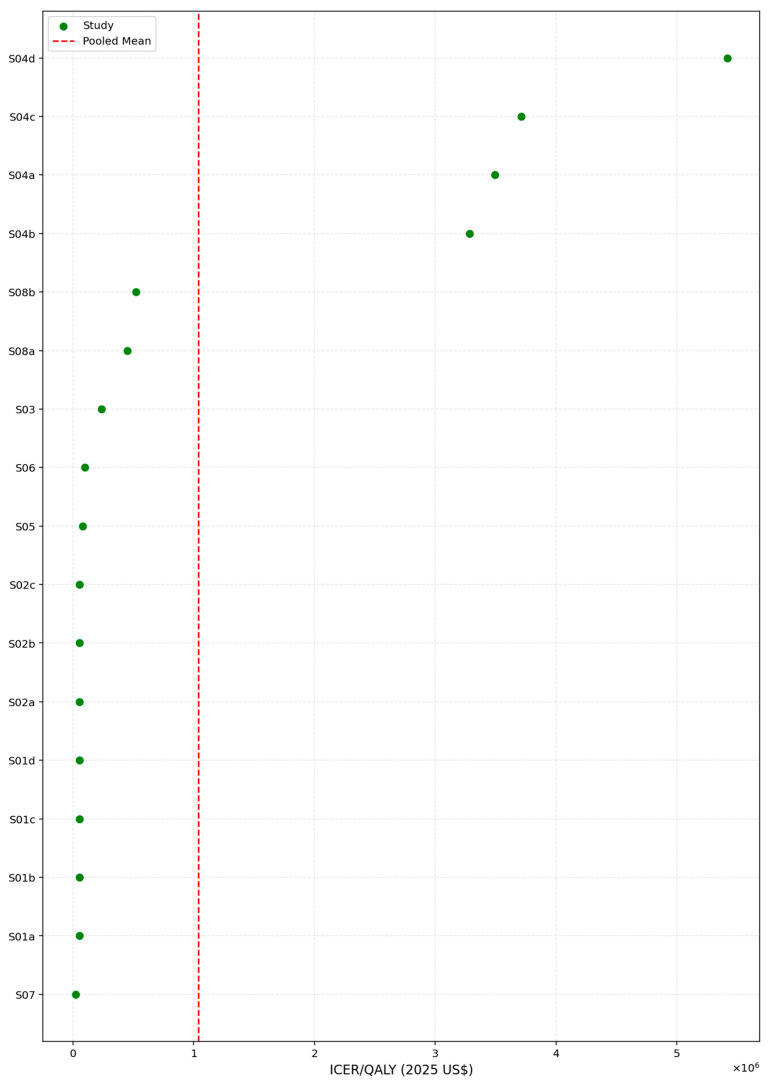
Cost-effectiveness ratio per study.

Using a log-transformed random-effects meta-analytic model, the pooled ICER per QALY in 2025 U.S. dollars was estimated at USD 211,596, with a 95% confidence interval ranging from USD 87,783 to USD 510,043. The model indicated substantial between-study variability, reflected by an I^2^ value of 70.8% and a corresponding between-study variance (τ^2^) of 2.43 on the log scale, as illustrated in Figure 11.

To further assess the economic performance of lipid-lowering interventions, we examined the distribution of ICER values across all included studies. As shown in Table 14, the ICERs displayed a markedly right-skewed pattern, with estimates ranging from USD 23,366 to USD 5.42 million per QALY gained. The median ICER of USD 83,717 was substantially lower than the mean value of USD 1.04 million, highlighting the influence of a small number of extremely high-cost scenarios. The interquartile range further reflected this dispersion, with the 25th percentile at USD 53,150 and the 75th percentile at USD 524,741, underscoring considerable heterogeneity in cost-effectiveness outcomes across settings and modeling assumptions. To address the discrepancy between arithmetic and pooled ICER estimates, we clarify that the random-effects pooled ICER represents the central, policy-relevant estimate, whereas the arithmetic mean ICER is influenced by extreme outliers and is not used for decision interpretation.

This pronounced skew suggests that while many interventions fall within or near conventional willingness-to-pay thresholds (e.g., USD 100,000–USD 150,000/QALY), a subset of studies reported exceptionally high costs relative to health gains, which may stem from niche populations, limited effectiveness, or unfavorable pricing structures. The interquartile range (USD 53,149 to USD 524,740) further emphasizes the heterogeneity in cost-effectiveness across contexts.

Given this variability, the median ICER may serve as a more appropriate benchmark for evaluating the typical cost-effectiveness performance of interventions in this domain, particularly when used to inform policy recommendations or price negotiations.

**Figure 12:**
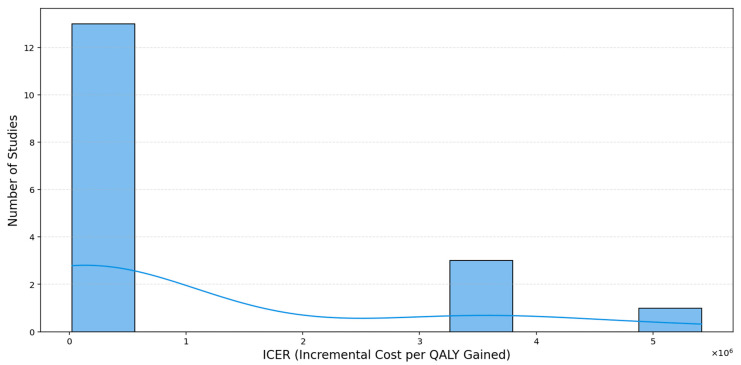
Distribution of ICER per QALY (USD).

**Figure 13:**
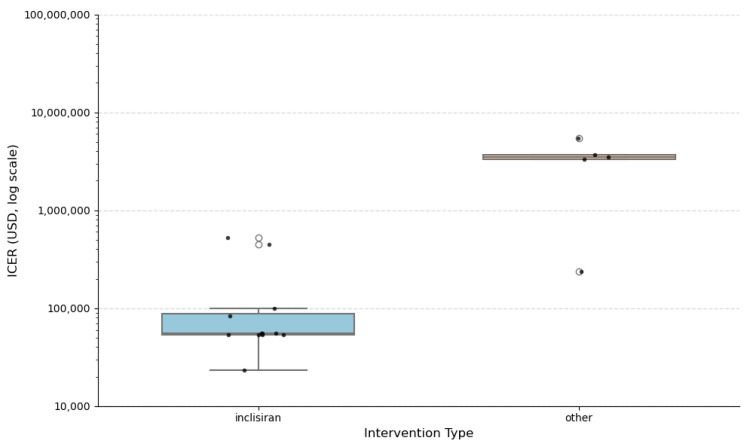
ICER per QALY by intervention type.

To further explore the distributional characteristics of cost-effectiveness across studies, a histogram and stratified boxplots of ICERs per QALY were constructed (Figure 11, Figure 12 and Figure 13).

The histogram (Figure 12) reveals a markedly right-skewed distribution, with a majority of ICER values clustered below USD 1 million per QALY, only a small number of studies extend into the multi-million-dollar range, and a single extreme case exceeds USD 5 million per QALY, creating a pronounced long right tail. The overlaid kernel density estimation (KDE) curve provides a smoothed representation of the underlying distribution, highlighting the concentration of studies at lower ICER values. This pattern underscores the importance of median-based measures when summarizing central tendencies, as the mean is highly sensitive to such extreme values.

Boxplots stratified by intervention type (Figure 13) clearly separates the cost-effectiveness of Inclisiran from other interventions. Inclisiran-based therapies cluster tightly at the lower end of the log-scaled ICER distribution (roughly USD 20,000–USD 100,000 per QALY), showing both lower costs and minimal variability. In contrast, the “other” interventions display a much broader and markedly higher ICER range, including several extreme outliers exceeding USD 1 million per QALY. The hollow circles represent statistical outliers (values beyond 1.5 times the interquartile range), while the overlaid black dots correspond to individual study-level ICER estimates, shown with slight horizontal jitter to improve visibility. This pattern indicates that Inclisiran consistently delivers more favorable and stable cost-effectiveness outcomes compared to alternative therapies.

**Figure 14:**
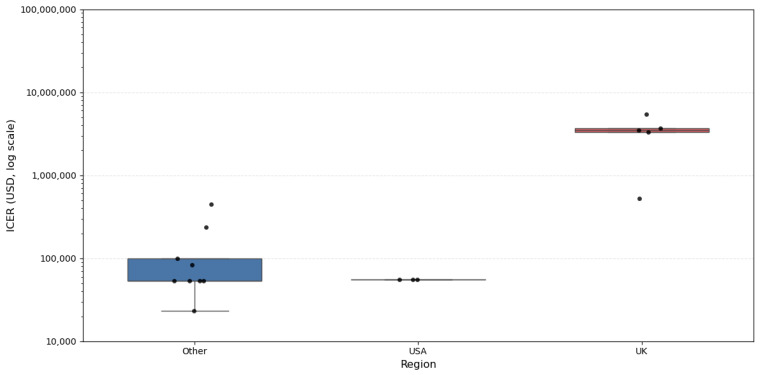
ICER per QALY by region.

Regional comparisons (Figure 14) reveal pronounced differences in cost-effectiveness across settings. Studies conducted in the United Kingdom display the highest and most widely dispersed ICERs, with several values exceeding USD 1 million, indicating markedly poorer economic performance. By contrast, ICERs from the USA and other regions cluster tightly at much lower levels, suggesting more consistent and comparatively efficient cost-effectiveness outcomes outside the UK. The overlaid individual data points illustrate this pattern by showing each study-level ICER directly.

### 3.8. Exploration of Variations in Cost-Effectiveness Ratios Across Studies

To evaluate the overall cost-effectiveness of lipid-lowering therapies relative to SoC, we conducted a series of meta-analyses estimating pooled NMB at varying WTP thresholds.

**Figure 15:**
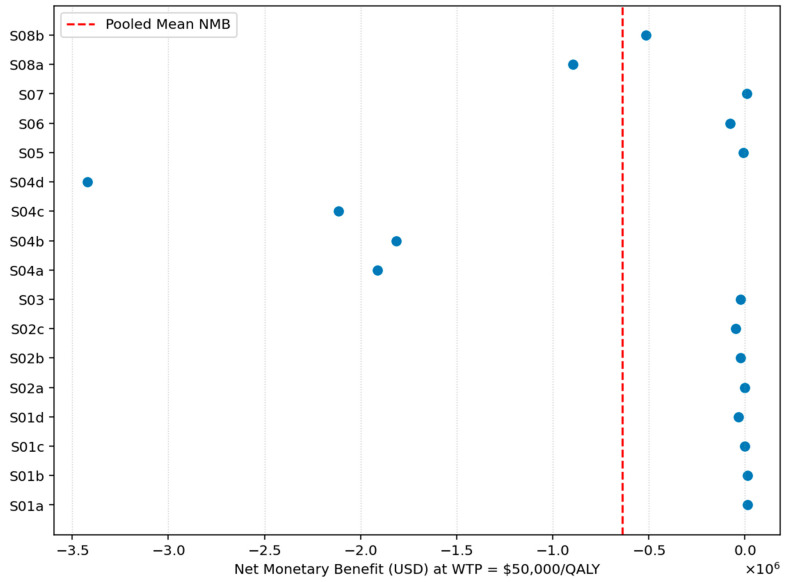
Net Monetary Benefit vs. standard of care at USD 50,000/QALY.

Figure 15 shows the study-level Net Monetary Benefit (NMB) estimates at a willingness-to-pay threshold of USD 50,000 per QALY. Most studies fall well below zero, and the pooled NMB line is also positioned clearly on the negative side of the axis. This pattern indicates that, on average, lipid-lowering interventions do not achieve positive monetary value relative to standard care at this WTP level. The consistently negative values imply that the additional costs of these interventions outweigh the monetized health gains under a USD 50,000 per QALY threshold.

**Figure 16:**
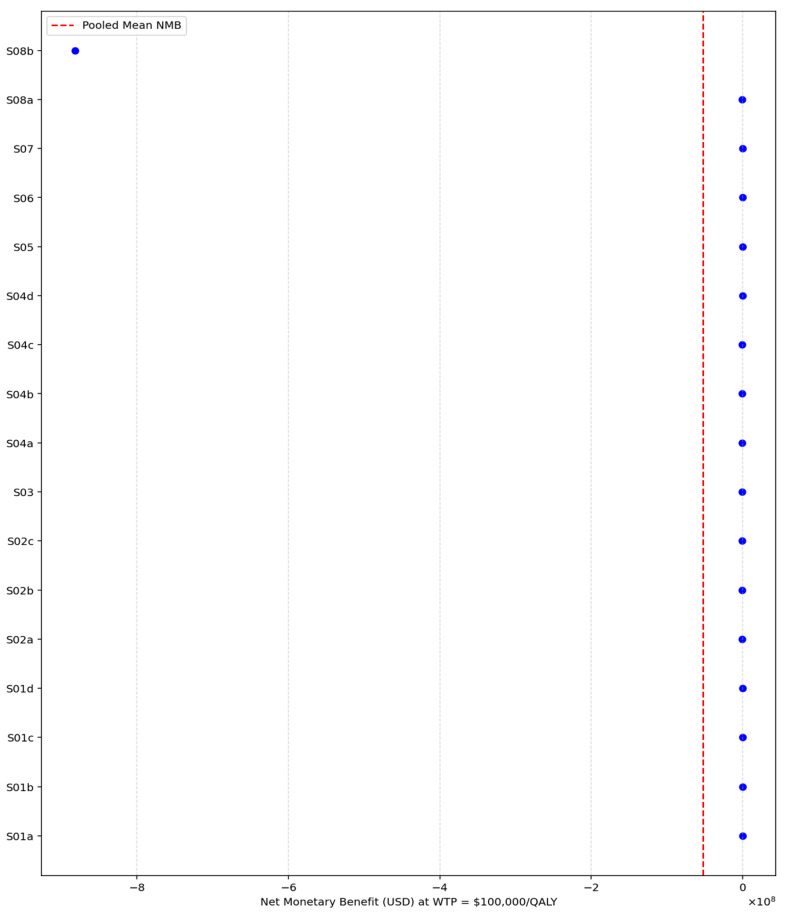
Net Monetary Benefit vs. standard of care at USD 100,000/QALY.

At the $100,000 per QALY threshold, shown in Figure 16, the pooled NMB remained strongly negative, with a mean value of −51,961,098.23 USD and a median of −96,871.70 USD. The range of NMB values was wide, spanning from −881,974,824 USD to USD 32,134, yet even the upper bound remained close to zero. The entire distribution remains dominated by large negative values, indicating that Inclisiran is not cost-effective relative to standard care even when the willingness-to-pay threshold is doubled. The concentration of NMB estimates below zero reinforces that higher monetary valuation of QALY gains does not materially alter the overall conclusion.

**Figure 17:**
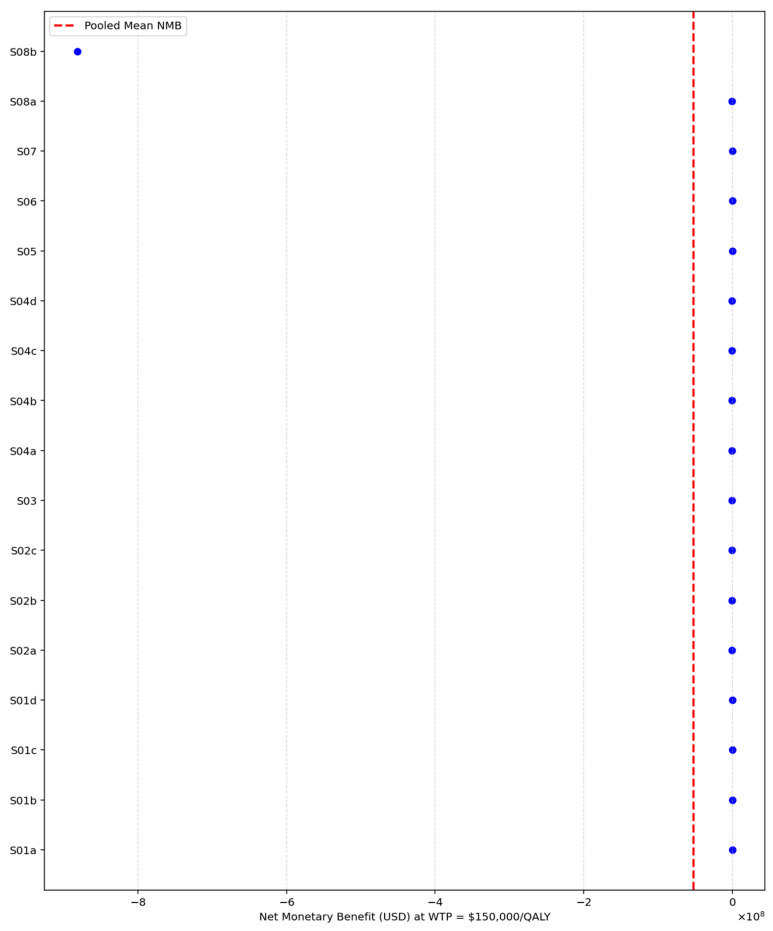
Net Monetary Benefit vs. standard of care at USD 150,000/QALY.

Finally, as illustrated in Figure 17, the pooled results again indicate a strongly negative economic profile, with a mean NMB of −51,944,095.73 USD and a median NMB of USD 89,567.36. Although the median shifts into positive territory at this higher willingness-to-pay level, the overall distribution remains dominated by substantial negative values, ranging from −881,964,992 USD to USD 79,134. This pattern shows only marginal improvement relative to lower thresholds and continues to demonstrate that, even under the most generous WTP assumptions commonly used in high-income settings, Inclisiran does not achieve cost-effectiveness on average when compared to standard care.

The substantial heterogeneity observed across studies reflects real differences in modeling frameworks, clinical populations, health-system prices, and WTP thresholds. Stratified results and meta-regression analyses confirm that these structural features meaningfully influence incremental costs and NMB values. Accordingly, pooled results should be interpreted as broad summaries across heterogeneous international settings rather than as a single universal cost-effectiveness estimate.

### 3.9. Meta-Regression

To explore temporal patterns in reported effectiveness, a meta-regression was performed examining the relationship between year of publication and QALY gain. The analysis yielded a slope estimate of 0.035 QALYs per year (*p* = 0.45), indicating no statistically significant trend over time in the magnitude of QALY gains across studies.

### 3.10. Publication Bias

Assessment of publication bias using Egger’s test revealed no significant asymmetry in the distribution of QALY gain estimates, suggesting a low likelihood of publication bias among the included studies.

## 4. Discussion

This meta-analysis evaluated the cost-effectiveness of Inclisiran, across multiple studies and settings. Overall, our findings reveal modest health gains in terms of QALYs, but highly variable and often unfavorable economic outcomes when compared to SoC. Mean ICER values frequently exceeded conventional WTP thresholds, particularly for high-cost agents such as Inclisiran, raising concerns about the broad affordability and sustainability of these interventions.

The evaluation of NMB across WTP thresholds of USD 50,000, USD 100,000, and USD 150,000 per QALY shows a clear upward trend in economic performance, though the overall results remain unfavorable at the pooled level. For Inclisiran, mean NMB values improved from −51.98 million USD at USD 50,000 to −51.94 million USD at USD 150,000. Nevertheless, the median NMB improved substantially, becoming positive at the highest threshold (USD 89,567), and maximum observed values also increased (up to USD 79,134), indicating that certain subgroups or contexts may achieve cost-effective outcomes when higher WTP assumptions are applied.

When stratified by intervention type, the contrast becomes clearer. Using the results from Table 3, Inclisiran demonstrates more favorable QALY gains (0.32 ± 0.30) and lower incremental costs relative to other lipid-lowering interventions. Although its average NMB remains negative at lower thresholds (e.g., −68,236 USD at USD 50,000), it becomes progressively less negative with increasing WTP and moves closer to cost-neutrality (e.g., −36,236 USD at USD 150,000). By comparison, therapies categorized as “Other” yield minimal QALY gains (0.03) and high incremental costs, producing consistently large negative NMB values (approximately −120,000 USD to −108,744 USD across thresholds).

Substantial regional variation was evident in the cost-effectiveness of lipid-lowering interventions. Across all WTP thresholds, studies conducted in the United Kingdom consistently produced the poorest economic outcomes, with extremely high incremental costs translating into large negative NMB values, even at the USD 150,000/QALY level. By contrast, economic evaluations from the United States, Singapore, and Australia showed more moderate costs and higher QALY gains, resulting in NMB estimates that were less negative and, in some cases, approaching or exceeding zero at higher WTP thresholds. These patterns indicate that certain health systems, especially those with greater investment capacity and more favorable pricing structures, may be better positioned to achieve cost-effective implementation of Inclisiran, whereas settings with higher intervention costs or more conservative cost-effectiveness thresholds continue to face substantial financial barriers. These findings highlight the importance of aligning reimbursement decisions with local pricing frameworks, clinical practice norms, and national budget constraints. In accordance with our study, recently published papers [28,29] demonstrate that among the eight major economic zones in China, there are intra-regional disparities due to the different population density and its effect on production efficiency. Moreover, the projected lifetime healthcare expenditure in China was one of the greatest, after the Indians, indicating a substantial economic burden. Thus, authorities should promote sustainable and balanced regional development, thus improving the way the government allocates the healthcare resources, which reduces the regional disparities and creates fairer health policies. Of course, all of the healthcare resources and the different types of treatment are very well weighted, in order to evaluate the cost-effectiveness outcomes and the health benefit that could be obtained after their administration.

Age-stratified analyses further illustrated the variability in economic value. Older populations (>60 years) derived the greatest health benefit (QALY gain = 0.32) and achieved the most favorable NMB profile (−36,236 USD at USD 150,000). Baratta et al. [30] recently published an article that evaluated the benefits of hypolipemiant treatment in elderly people, discussing the latest published international guidelines. The authors declared that both young and elderly patients with ASCVD should be treated the same, while patients that are over 75 years old and are at high cardiovascular risk or above should have initiated a moderate-intensity statin, with the possibility to increase the doses in further evaluations. Rosada et al. share the same perspective as Baratta et al., affirming the need for consistent and sustainable screening and treatment in elderly hyperlipidemic patients [31]. In contrast, patients under 45 years showed negligible health gains (QALY gain = 0.03) and prohibitively high incremental costs (mean = USD 1.98 million), resulting in sharply negative NMBs. These results underscore the need for targeted use of lipid-lowering therapies in older, high-risk populations where the benefit-to-cost ratio is more favorable. Contrary to the results obtained by our study on Inclisiran, studies published on statins demonstrate that they are highly or intermediate cost-effective on values of LDL-C over 130 mg/dL [32].

The consistent trends observed across both ICER- and NMB-based analyses reinforce the robustness of our findings. However, substantial heterogeneity persists across studies, particularly those conducted in the UK. These variations may reflect methodological differences, changes in clinical guidelines, or shifts in pharmaceutical pricing. Importantly, the divergence between mean and median NMB values suggests that aggregate analyses may obscure cost-effectiveness in specific subgroups, supporting the use of distributional statistics in economic evaluation.

Inclisiran has demonstrated robust efficacy with a favorable safety profile and the advantage of infrequent, vaccine-like administration, which enhances patient adherence. Nevertheless, its high cost restricts widespread use, underscoring the need for price renegotiation and targeted reimbursement models to improve accessibility. Our findings confirm that the cost-effectiveness of Inclisiran is context-dependent, shaped by population risk and system-level capacity, which limits its adoption for primary prevention under current conditions.

Beyond its clinical impact, Inclisiran illustrates how innovative biologics can progress efficiently from laboratory to clinical practice when supported by appropriate infrastructure and multidisciplinary expertise. This translational trajectory is directly relevant to the Cantavac2.0 project, where our meta-analysis will serve as an example of how vaccine-like biologics, rarely administered yet providing long-term efficacy, can guide the development and evaluation of novel prophylactic and therapeutic products within a national and European framework for biotechnological autonomy.

## 5. Strengths and Limitations

This meta-analysis has several limitations: only studies conducted in adult populations from high-income countries were included, the number of eligible articles was limited, and the high cost of Inclisiran currently restricts widespread use. Moreover, cross-country comparisons are not entirely uniform, as treatment is not administered in populations with comparable cardiovascular risk according to SCORE stratification. Nonetheless, a key strength of this work is that it represents the first meta-analysis to evaluate the cost-effectiveness of Inclisiran, prioritizing its proven clinical efficacy while contextualizing cost considerations. As some studies contributed multiple stratified sub-scenarios, this may increase their representation within the dataset; however, these sub-cohorts were treated as separate modeled populations, and results are interpreted with this context in mind.

## 6. Conclusions

Taken together, our findings suggest that the cost-effectiveness of Inclisiran is highly context-dependent, shaped by local pricing, population risk, and system-level capacity. While Inclisiran demonstrates potential economic value in high-income settings or among high-risk patients, its widespread adoption for primary prevention appears unjustified under current conditions. Policymakers should consider risk-based targeting, price renegotiation, and performance-based reimbursement models to improve the value proposition of such interventions.

Future research should focus on long-term real-world effectiveness, refined modeling of budget impact, and comparative evaluations that account for alternative lipid-lowering strategies. Such efforts are essential to inform nuanced reimbursement decisions and ensure that resource allocation aligns with both clinical value and economic sustainability.

## Figures and Tables

**Table 1:** General characteristics of included studies.

Study ID	Authors	Year	Country	Treatment	Model	No. Patients	Age (avg)	Gender (Female)	Diabetes
S01a	Lim et al. [21]	2025	Singapore	ASCVD Inclisiran + SoC	Lifetime Markov model	ORION-11	-	-	-
S01b	Lim et al. [21]	2025	Singapore	Sec Prev HeFH Inclisiran + SoC	Lifetime Markov model	ORION-9	59.26	39.1%	16.50%
S01c	Lim et al. [21]	2025	Singapore	PPER Inclisiran + SoC	Lifetime Markov model	ORION-10	62.28	54.4%	65.80%
S01d	Lim et al. [21]	2025	Singapore	Prim Prev HeFH Inclisiran + SoC	Lifetime Markov model	ORION-9	52.36	45.0%	9.50%
S02a	Desai et al. [22]	2022	USA	ASCVD $50,00 WTP Inclisiran + SoC	Lifetime Markov model	3660	66.00	31.0%	45.00%
S02b	Desai et al. [22]	2022	USA	ASCVD $100,000 WTP Inclisiran + SoC	Lifetime Markov model	3660	66.00	31.0%	45.00%
S02c	Desai et al. [22]	2022	USA	ASCVD $150,000 WTP Inclisiran + SoC	Lifetime Markov model	3660	66.00	31.0%	45.00%
S03	Galactionova et al. [23]	2022	Switzerland		open-cohort Markov model	17,024	71.00	40.0%	27.00%
S04a	Morton et al. [20]	2023	UK	Inclisiran age of intervention 30	Microsimulation	458,692	30.00	-	-
S04b	Morton et al. [20]	2023	UK	Inclisiran age of intervention 40	Microsimulation	456,016	40.00	-	-
S04c	Morton et al. [20]	2023	UK	Inclisiran age of intervention 50	Microsimulation	449,476	50.00	-	-
S04d	Morton et al. [20]	2023	UK	Inclisiran age of intervention 60	Microsimulation	434,024	60.00	-	-
S05	Zhou et al. [24]	2024	China	Inclisiran + statin	Markov model	121.00	53.26	30.58%	-
S06	Kam et al. [25]	2020	Australia	Inclisiran + statin	Markov cohort state-transition model	1000.00	66.00	-	-
S07	Wang et al. [26]	2025	China	Inclisiran	Markov model	27,564.00	inclusion age 40 to 85 years	-	-
S08a	Morton et al. [19]	2025	Australia	Inclisiran	Microsimulation	54 unique risk profiles, simulated 10,000 times	simulated data, inclusion age 40 to 85 years	-	-
S08b	Morton et al. [19]	2025	United Kingdom	Inclisiran	Microsimulation	54 unique risk profiles based on UK biobank, simulated 10,000 times	simulated data, inclusion age 40 to 85 years	-	-

**Table 2:** Characteristics of costs, QALY, and ICER from included studies.

Study ID	Authors	Year	Country	Treatment	QALY Gain	Total QALY	PPP-Adjusted ICER/QALY USD	PPP-Adjusted Total Cost USD	PPP-Adjusted ICER USD
S01a	Lim et al. [21]	2025	Singapore	ASCVD Inclisiran + SoC	0.79	8.33	53,149.66	51,124.11	27,534.38
S01b	Lim et al. [21]	2025	Singapore	Sec Prev HeFH Inclisiran + SoC	0.94	10.29	53,149.66	61,865.18	35,527.60
S01c	Lim et al. [21]	2025	Singapore	PPER Inclisiran + SoC	0.65	11.77	53,149.66	48,117.04	34,527.11
S01d	Lim et al. [21]	2025	Singapore	Prim Prev HeFH Inclisiran + SoC	0.32	16.41	53,149.66	62,429.68	51,287.21
S02a	Desai et al. [22]	2022	USA	ASCVD $50,00 WTP Inclisiran + SoC	0.43	9.84	55,421.87	154,067.36	23,051.88
S02b	Desai et al. [22]	2022	USA	ASCVD $100,000 WTP Inclisiran + SoC	0.43	9.84	55,421.87	176,040.53	46,097.32
S02c	Desai et al. [22]	2022	USA	ASCVD $150,000 WTP Inclisiran + SoC	0.43	9.84	55,421.87	199,085.97	69,142.77
S03	Galactionova et al. [23]	2022	Switzerland		0.291	11.42	235,403.33	134,827.35	37,402.95
S04a	Morton et al. [20]	2023	UK	Inclisiran age of intervention 30	0.035	20.64	3,494,536.89	123,542.66	1,913,544.06
S04b	Morton et al. [20]	2023	UK	Inclisiran age of intervention 40	0.034	18.35	3,286,519.03	113,387.86	1,815,767.61
S04c	Morton et al. [20]	2023	UK	Inclisiran age of intervention 50	0.027	15.54	3,710,957.69	99,571.70	2,115,993.11
S04d	Morton et al. [20]	2023	UK	Inclisiran age of intervention 60	0.015	12.15	5,417,450.97	81,003.76	3,422,471.51
S05	Zhou et al. [24]	2024	China	Inclisiran + statin	0.21	9.42	83,717.19	23,715.92	17,675.23
S06	Kam et al. [25]	2020	Australia	Inclisiran + statin	0.468	7.40	100,127.64	46,957.23	99,544.70
S07	Wang et al. [26]	2025	China	Inclisiran	0.374	7.77	23,366.74	13,259.00	8739.16
S08a	Morton et al. [19]	2025	Australia	Inclisiran	0.1402	15.41	450,256.67	533,269.62	902,919.79
S08b	Morton et al. [19]	2025	United Kingdom	Inclisiran	0.19665	18.20	524,740.92	881,994,489.92	524,740.92

**Table 3:** Analysis of variation in cost-effectiveness by intervention type.

WTP (USD/QALY)	Intervention	QALY Gain ± SD	Incremental Cost ± SD (USD)	NMB Mean (USD)
50,000	Inclisiran	0.32 ± 0.30	84,211 ± 209,703	−68,236
	Other	0.291	134,827	−120,277
100,000	Inclisiran	0.32 ± 0.30	84,211 ± 209,703	−52,236
	Other	0.291	134,827	−5.766
150,000	Inclisiran	0.32 ± 0.30	84,211 ± 209,703	−36,236
	Other	0.0291	134,827	−108,744

**Table 4:** Analysis of variation in cost-effectiveness by region.

Country	QALY Gain (Mean ± SD)	Incremental Cost (USD)	Net Monetary Benefit (USD)
Australia	0.30 ± 0.23	501,232 ± 568,072	−486,027 ± 579,661
Singapore	0.67 ± 0.26	37,121 ± 10,054	−3371 ± 22,270
USA	0.43 ± 0.00	47,271 ± 23,632	+17,229 ± 23,632
Switzerland	0.29	37,403	−$22,853
China	0.29 ± 0.12	13,207 ± 6319	−1393 ± 12,117
UK	0.03 ± 0.01	2,316,944 ± 747,547	−2,315,557 ± 747,993
United Kingdom	0.028 ± 0.01	1,958,503.44 ± 1,030,300.36	−2,315,556.57 ± 747,993.41

**Table 5:** Analysis of variation in cost-effectiveness by age group.

Age Group	QALY Gain	Incremental Cost (USD)	NMB (USD)
<45	0.03 ± 0.00	1,984,976 ± 52,043	−1,983,251 ± 52,018
45–60	0.37 ± 0.20	596,437 ± 552,082	−577,724 ± 558,018
>60	0.40 ± 0.09	652,727 ± 598,240	−632,535 ± 602,141

**Table 6:** Inclisiran specific summary.

Metric	Mean	Median	Min	Max
Incremental Cost (USD)	655,621.96	51,287.21	8739.16	3,422,471.51
QALY Gain	0.35	0.35	0.02	0.94
ICER (USD/QALY)	1,041,525.96	83,717.19	23,366.74	5,417,450.97

**Table 7:** NMB for Inclisiran vs. SoC.

WTP Threshold	Mean NMB (USD)	Median NMB	Min NMB	Max NMB
50,000	−51,978,100.73	−98,221.70	−881,984,657	5441
100,000	−51,961,098.23	−96,871.70	−881,974,824	32,134
150,000	−51,944,095.73	89,567.36	−881,964,992	79,134

**Table 8:** Cost-effectiveness classification table.

WTP Threshold (USD/QALY)	Cost-Effective (ICER)	Cost-Effective (NMB)	Total Studies
50,000	3	3	17
100,000	5	5	17
150,000	8	8	17

**Table 9:** Country comparison for Inclisiran affordability.

Country	Mean ICER (USD/QALY)	WTP Threshold (USD/QALY)	Avg. Country NMB (USD)	No. of Studies	No. Cost-Effective	Can Afford Inclisiran?
Singapore	53,149.66	50,000	−3371.00	4	0	No
USA	55,421.87	150,000	+17,229	3	3	Yes
China	53,541.97	30,000	−1393	2	1	No
UK	3,977,366.15	30,000	−2,315,557	4	3	No
Australia	275,192.16	N/A	N/A	2	0	Unknown
Switzerland	235,403.33	N/A	−22,853	1	0	Unknown
UK (2nd entry)	524,740.92	N/A	−2,315,556	1	0	No

**Table 10:** OLS meta-regression model of Net Monetary Benefit by country and intervention type at a USD 50,000/QALY threshold.

Covariate	Coefficient Estimate (USD)	*p*-Value	Interpretation
Intercept	396,796,900	0.025	Baseline NMB (reference: Australia, Inclisiran)
Year	−196,046,100	0.025	NMB declines over time
China	+392,363,300	0.1428	Higher NMB vs. Australia
Singapore	+196,419,900	0.3720	Higher NMB vs. Australia
Switzerland	−391,816,600	0.2203	Lower NMB vs. Australia
UK	−293,711,100	0.1681	Lower NMB vs. Australia
USA	−391,851,200	0.1526	Lower NMB vs. Australia
Inclisiran + Statin	−597,896,300	0.0765	Lower NMB vs. Inclisiran

**Table 11:** OLS meta-regression model of Net Monetary Benefit by country and intervention type at a USD 100,000/QALY threshold.

Predictor	Coefficient (USD)	*p*-Value	Interpretation
Intercept	342,325,300	0.0028	Baseline NMB (reference Australia, Inclisiran)
Year	−169,390,300	0.0028	NMB decreases over time
China	+605,419,400	0.0106	Significantly higher NMB vs. reference country
Singapore	+690,115,500	0.0038	Significantly higher NMB vs. reference
Switzerland	+181,827,200	0.4134	No significant difference
UK	+242,603,100	0.1366	Higher NMB but not significant
USA	+181,799,500	0.2808	Higher NMB but not significant
Inclisiran + Statin	0	-	No measurable difference

**Table 12:** OLS meta-regression model of Net Monetary Benefit by country and intervention type at a USD 150,000/QALY threshold.

Predictor	Coefficient (USD)	*p*-Value	Interpretation
Intercept	342,328,200	0.0028	Baseline NMB (reference: Australia, Inclisiran)
Year	−169,391,800	0.0028	Statistically significant downward trend in NMB
China	+605,421,700	0.0106	Significantly higher NMB
Singapore	+690,137,600	0.0038	Significantly higher NMB
Switzerland	+181,825,800	0.4134	Not significant
UK	+242,592,300	0.1366	Not significant
USA	+181,805,100	0.2807	Not significant
Inclisiran + Statin	0	-	No measurable difference

**Table 13:** Pooled incremental cost and QALY gain of lipid-lowering interventions compared to standard of care.

Metric	Value
Pooled Incremental QALY Gain	0.349 QALYs per patient
95% CI for QALY Gain	(0.213, 0.485)
Pooled Incremental Cost	USD 33,416,186.31
95% CI for Incremental Cost	(−31,898,048.87 USD, USD 98,730,421.49)

**Table 14:** ICER/QALY summary.

Statistic	Value (USD per QALY)
Minimum	23,366.74
25th Percentile	53,149.66
Median (50th Percentile)	83,717.19
Mean	1,041,526
75th Percentile	524,740.92
Maximum	5,417,450.97
Standard Deviation	1,736,796

## Data Availability

The data presented in this study are available on request from the corresponding author.
